# Primacy of Human Odors Over Visual and Heat Cues in Inducing Landing in Female *Aedes aegypti* Mosquitoes

**DOI:** 10.1007/s10905-022-09796-2

**Published:** 2022-05-23

**Authors:** Benjamin D. Sumner, Ring T. Cardé

**Affiliations:** Department of Entomology, University of California, Riverside, CA 92521 USA

**Keywords:** Anemotaxis, carbon dioxide, landing, odor plume, orientation, wind tunnel

## Abstract

Although human skin odor is thought to be the cue that anthropophilic mosquitoes use to discriminate us from other potential hosts, the precise details of how they use skin odor to find and land on a human is unclear. We found that *Aedes aegypti* land on a source of skin odor without a co-located visual cue. By collecting human odor on glass beads and using identical glass beads to visually conceal skin odor and heat cues, we were able to study mosquito landing on skin odor, heat, and visual cues separately. Landing is necessary for blood feeding which is a required behavior for the *Aedes aegypti* life cycle as well as the behavior responsible for the epidemiological impact of mosquitoes. Therefore, we consider it to be the diagnostic measure of the importance of a host cue. In two-choice tests, a skin odor source had the highest valence for landing, followed by a combination of heat and a visual cue, and finally heat and visual cues presented separately. We also measured the durations of the landings, though no significant differences were found.

**Supplementary Information:**

The online version contains supplementary material available at 10.1007/s10905-022-09796-2.

## Introduction

Female *Aedes aegypti* mosquitoes vector dengue, Zika, chikungunya, and yellow fever viruses by repetitive feeding on humans. Skin odor is thought to be the cue that female *Ae. aegypti* and other anthropophilic mosquitoes use to discriminate humans from other endothermic vertebrates (Gouck 1972; Takken et al. 1997; Dekker et al. 2001, 2002; Besansky et al. 2004; McBride 2016). *Aedes aegypti*, following an encounter with an above-ambient concentration of CO_2_, use skin odor to pinpoint a landing site suitable for a blood meal.

Due to the special role of skin odor, we presented *Ae. aegypti* with skin odor without co-located visual cues. We also quantified landing, a behavior necessary for mosquito blood feeding. We found that *Ae. aegypti* landed on a source of skin odor presented without a co-located visual cue more frequently than on the visual cue, a heat cue, or even a heated visual cue. The primacy of skin odor contrasts with the view that this diurnal mosquito relies primarily on visual cues during host seeking after navigating upwind along a CO_2_ plume (van Breugel et al. 2015). Unlike prior work with mosquitoes and other insects (Goodman 1960; Srinivasan and Zhang 1997; Srinivasan et al. 2000; van Bruegel and Dickinson 2012; Parker et al. 2015), in which visual cues elicited landing or persistent nearby flight, we presented heat and skin odor without co-located visual cues. Mosquitoes nonetheless landed on both visually indistinct source of skin odor and, less frequently, heat stimuli.

To assess the relative valence of host-seeking cues used by female *Ae. aegypti*, we presented skin odor, heat and visual cues in a free-flight wind tunnel, which allowed us to separate a CO_2_ plume from other host cues. Naïve *Ae. aegypti* were first exposed to an above-ambient concentration of CO_2_ at the tunnel’s downwind end and then offered a choice of two competing stimuli on the wind-tunnel floor 50 cm upwind of the release cage. This choice allowed us to determine which cues host-seeking *Ae. aegypti* preferred. We also tallied the durations of the landings but found no significant differences across cue combinations.

## Materials and Methods

### Insects

We used the “Orlando” strain of *Ae. aegypti* (Kuno 2010). The colony was maintained in a L:D 14:10 h cycle, at 25 °C and 70% RH in the UCR Insectary and Quarantine Facility. The females used for colony maintenance were fed defibrinated bovine blood through an artificial membrane (HemoStat Laboratories, Dixon, CA, USA). Larvae were reared in plastic containers and fed TetraMin Tropical Tablets (Tetra Holding GmbH, Melle, Germany). Approximately 50 larvae were reared in each container. All pupae (male and female) from three containers were allowed to emerge into screen cages (BugDorm 30 × 30 × 30 cm, MegaView Science Co., Ltd., Taichung, Taiwan) containing 10% (v) sucrose solution provided *ad libitum*. All mosquitoes were assumed to have mated and were used only once. Five female mosquitoes, 3–9 days post eclosion, were transferred to cylindrical acrylic release cages (7 × 8 cm i.d.) three hours before the start of assays. *Aedes aegypti* were assayed 4–8 h into their photophase.

### Wind Tunnel

**Fig. 1:**
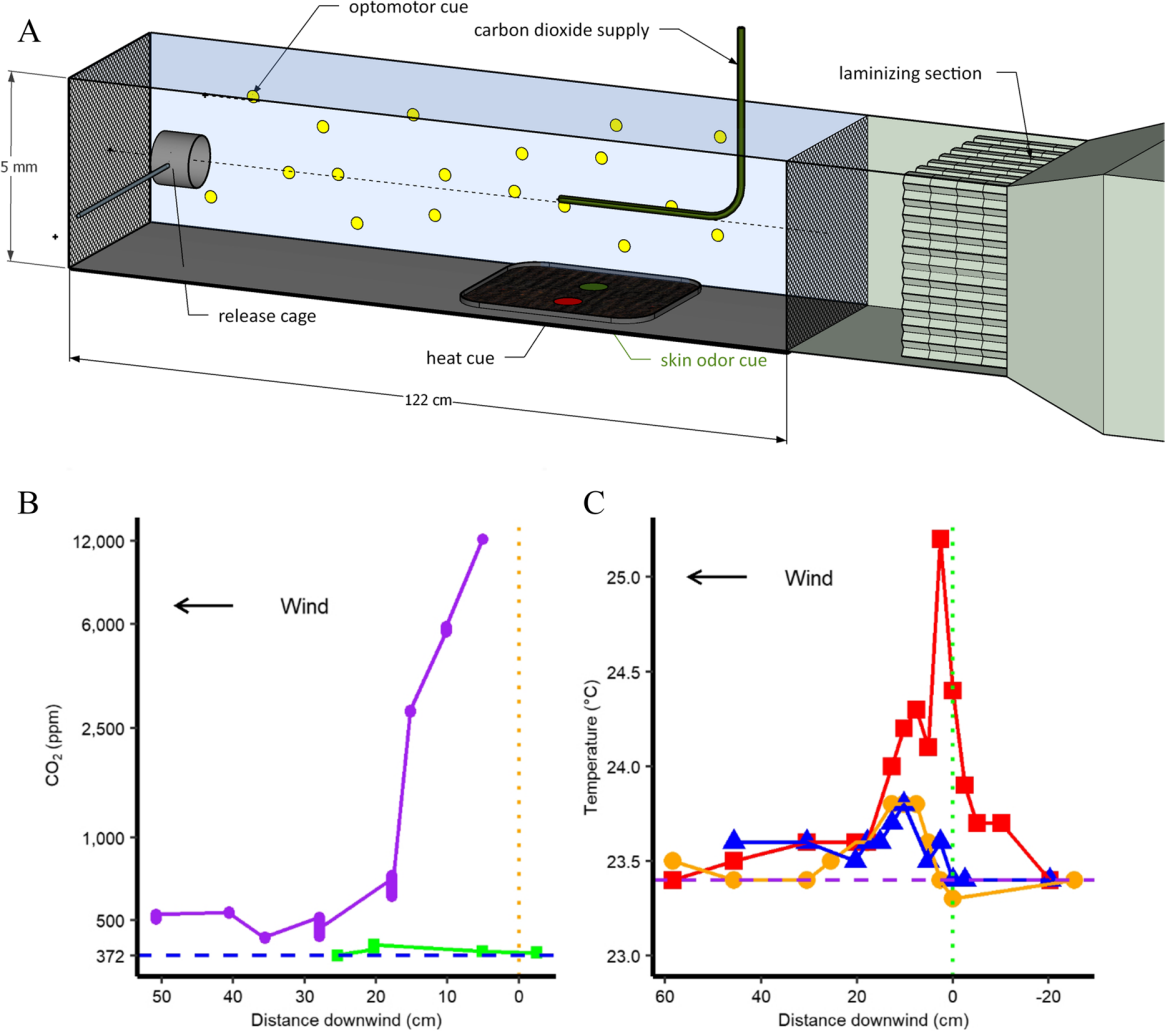
(**A**) Diagram of wind tunnel. Wind flow is from right to left. (**B**&**C**) The x-axes go right to left, to match the physical arrangement of the wind tunnel. (**B**) Carbon dioxide concentration in parts per million (ppm) centered in the tunnel at a height of 14 cm (purple circles) and 1 cm (green squares). The dashed horizontal blue line represents concurrent measurements of the ambient CO_2_ concentration. (**C**) The air temperature downwind of the heat pad at three heights. A height of 1 cm is shown in red (squares), 2 cm in orange (circles), and 3 cm in blue (triangles). Concurrent measurements of the ambient air temperature are shown in purple

The flight and landing of mosquitoes were observed in a glass wind tunnel 122 × 30.5 × 30.5 cm (Fig. 1A). The exterior of the wind-tunnel floor was covered with black construction paper. Yellow tape was applied to the outside of the glass sidewalls in “x” patterns to provide optomotor feedback (shown only on one side in Fig. 1A). Additional visual feedback was available from the wind tunnel’s structural components and the room external to the wind tunnel. The mosquitoes were therefore not in a featureless visual surround. Air was drawn into the wind tunnel from an adjacent uninhabited room (25 °C and 70% RH). The experimenter did not breathe while loading the release cage into the wind tunnel each trial, so that mosquitoes being transferred into the tunnel were not exposed to a human exhalation of CO_2_. Airspeed throughout the wind tunnel was 0.2 m/s. The mosquitoes were recorded for 6 min using a video camera (ICD 48, 6 mm lens; Ikegami, Maywood, NJ, USA) positioned 50 cm above the wind tunnel. This allowed observation of most of the tunnel, including the entire area in which cues were presented. Illumination was provided by four infrared LED light banks (AXIS T90A, 850 nm, Axis Communications AB, Lund, Sweden) mounted behind a stainless-steel screen at the downwind end of the wind tunnel. Diffuse room light, provided by incandescent bulbs, measured at ~ 14 lux in the tunnel. The visible spectrum lights were aimed at the junction of the wall and ceiling opposite the wind tunnel. Luminance was measured from a point centered in the wind tunnel and 70 cm from its upwind end, with a Gossen Ultra-Pro (GOSSEN GmbH, Nuremberg, Germany). The luminance at the downwind end was 4 cd/m^2^, the upwind end 1 cd/m^2^, the room 4 cd/m^2^, the wall 8 cd/m^2^, and the beads 0.067 cd/m^2^.

A visually indistinguishable layer of beads allowed heat or skin odor to be presented independent of a co-located visual cue. The two cue presentation areas in each trial were arranged in the middle of the aluminum pan 13.5 cm from the upwind and downwind ends of the aluminum pan and with 5 cm separating each cue from each other and from the lateral edges of the pan (Fig. 1A). This consistency of location was crucial because the beads were not visibly distinguishable. The pan was placed with its downwind edge 70 cm from the downwind.

To simulate the presence of an upwind vertebrate host, 100 ml/minute of CO_2_ at 4% concentration mixed with tank air was carried to the wind tunnel via a 3-m-long Tygon® tube, ensuring temperature equilibration. The tube was connected to a glass, L-shaped tube (OD 5.5 mm, ID 3.5 mm) that descended 15 cm from the ceiling on the wind tunnel and extended 20 cm downwind to 60 cm upwind from the release cage. The 4% CO_2_ mix exited the inner opening at ~ 0.4 m/s, but there was no detectable difference in airspeed 1 cm downwind of the CO_2_ release point (Omega HHF 52 anemometer, Omega Engineering, Inc., Stamford, CT, USA) and the temperature was identical to the air in the wind tunnel (to within 0.1 °C, same device). The CO_2_ release tube was centered so that the generated plume of CO_2_ would engulf the release cage. The CO_2_ plume was turbulent enough to produce the distinct packets of CO_2_ needed to elicit upwind flight (Dekker and Cardé 2011) yet compact enough not to mingle with the skin odor plume below. The CO_2_ plume structure was verified with a visible “smoke” plume of titanium dioxide and hydrochloric acid produced by the reaction of TiCl_4_ with damp air.

We measured the CO_2_ concentration with a GasHound CO_2_ detector (Model LI-800, LI-Core, Nebraska, USA) at 10 points in line with the CO_2_ source as well as 10 points along the floor (Fig. 1B). Carbon dioxide concentrations were recorded after one minute of equilibration. However, because the gas being sampled is drawn through a tube, a pump, and a filter, this instrument produces time-averaged values in contrast to the nearly instantaneous sensing of CO_2_ by mosquitoes (Dekker and Cardé 2011). The dashed blue line in Fig. 1B shows that the CO_2_ did not extend to within 1 cm of the floor 25 cm downwind of the other cue presentation areas. The heights of CO_2_ measurements refer to height above the beads. The height was kept the same for measurements downwind of the beads. For measurements downwind of the beads this means the true height was 0.7 cm above the tunnel floor. To land on a floor cue the mosquito must fly both vertically down and upwind of the CO_2_ source. A few mosquitoes approached the glass CO_2_ release tube. Some cast a few centimeters downwind for several seconds, and others briefly landed on the tip of the CO_2_ release tube, but they were not scored.

The fluctuating concentration of CO_2_ at the release cage was expected to elicit take off and to sensitize the mosquitoes to other cues. Because CO_2_ flux occurs in the field, we did not conduct a CO_2_ free control. If we had eliminated CO_2_, the lower take off rate would have made the sample size small for a reason that is not biologically meaningful. The separation of the CO_2_ plume vertically from the skin odor and heat plumes showed that the cues do not need to be encountered simultaneously.

**Fig. 2:**
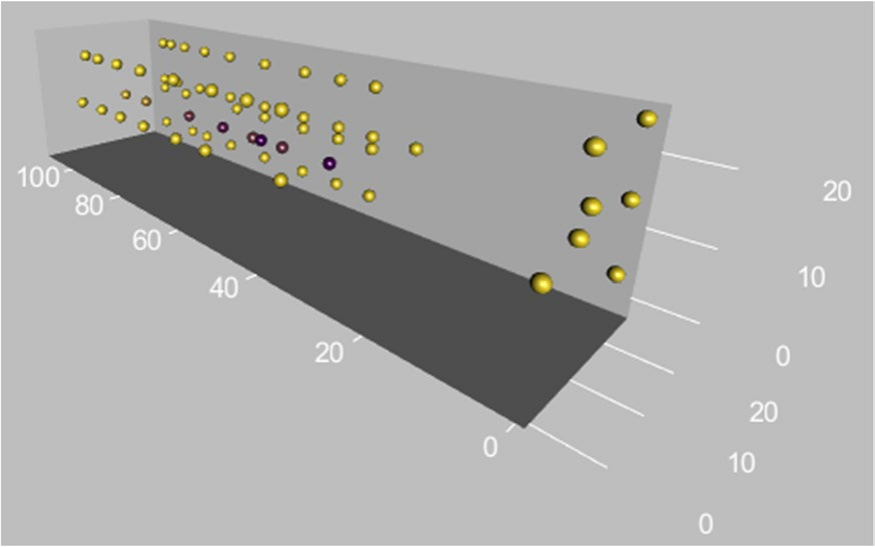
Plot of the concentration of carbon dioxide at several locations in the wind tunnel under assay conditions. The color of each dot varies from light yellow (406 ppm) to dark purple (4860 ppm), with intermediate concentrations shown in shades of yellow mixed with purple. The lack of dark purple dots anywhere other than directly downwind of the CO_2_ release point shows the separation of the cues available to the mosquito at any point in its flight. The x-axis shows the distance downwind from the upwind end of the tunnel in centimeters. The y- and z-axes show the displacement from the floor and room-side tunnel wall in centimeters

To ensure that elevated concentrations of CO_2_ were not present in other parts of the wind tunnel, we took additional readings with a portable Amprobe CO2-100 m (Amprobe, Everett, WA, USA). The readings are shown graphically in Fig. 2.

### Cues Presented on the Floor

The orientation of all cues presented on the floor was assigned randomly at the start of each day. To account for possible position effects, their position was switched halfway through the assays being conducted of that type on that day. The beads were put in the same place each time.

### Odor Cue

Human skin odor was collected onto black glass beads (12/0 Czech Glass Seed, approximately 2 mm OD toroidal, Precosia Ornela, Zásada, Czech Republic)) by placing 25 ml of beads into a polyester/cotton blend sock, which was then worn by a volunteer (two males and one female) for three hours (Bernier et al. 1999). One volunteer was used per day. Volunteers refrained from alcohol, spicy foods, vigorous exercise, and scented products for three days before and while wearing the beads. The unavoidable variation in human odor on a day-to-day basis was accounted for by statistical blocking.

**Fig. 3:**
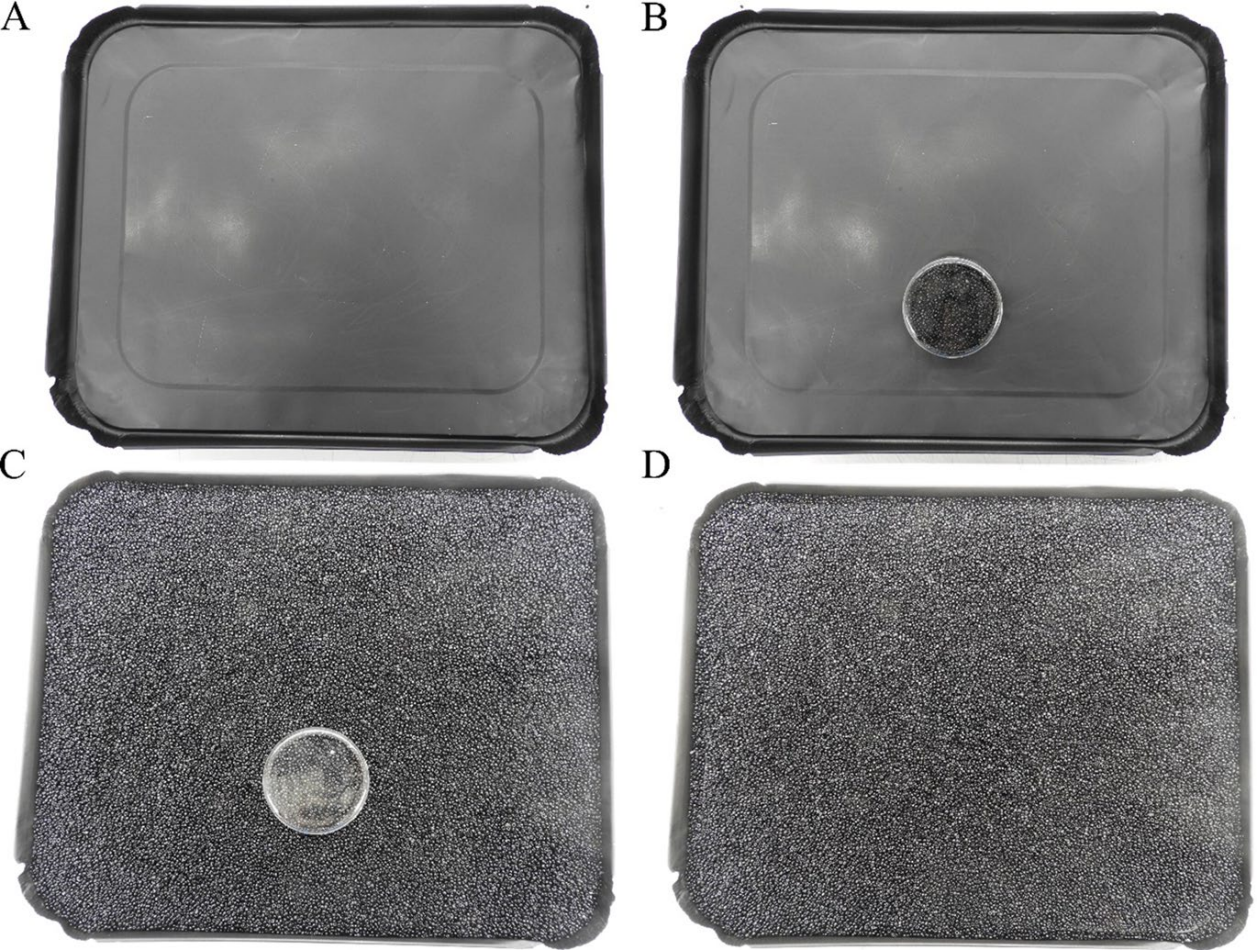
Odor cues were presented on a 5-mm layer of black glass beads (12/0 Czech Glass Seed, approximately 2 mm OD toroidal, Precosia Ornela, Zásada, Czech Republic) in a 32.5 by 26.5 cm black aluminum pan. (**A**) The empty aluminum pan. (**B**) The aluminum pan with a Petri dish containing odor beads. (**C**) The aluminum pan with inverted Petri dish containing odor beads surrounded by clean beads without odor. (**D**) Petri dish removed leaving no visual target of odor beads within the clean beads

The beads treated with skin odor were poured into a 55-mm-diameter plastic Petri dish. Beads were allowed to air dry for one hour before assays. The bead-filled Petri dish was then covered with a black aluminum pan (Fig. 3A). While pressing the Petri dish into the pan, the whole arrangement was flipped (Fig. 3B). Clean beads, otherwise identical to those used to collect odor, were then poured into the aluminum pan (Fig. 3C). Removal of the Petri dish produced a visually indistinguishable patch of beads treated with skin odor surrounded by clean beads (Fig. 3D). The area of clean beads within which landing was scored was equal in size (55 mm diameter) to the odoriferous beads. It was crosswind and opposite to the odoriferous beads.

Between use, beads were sonicated with detergent (Micro-90; Aldrich; St. Louis MO, USA), rinsed with distilled water, rinsed with acetone, and baked at 200 °C for 8 h. One week before assays were conducted, the aluminum pans used to hold the beads (32.5 × 26.5 × 1.6 cm, Catering Tray Lid, Smart & Final, Commerce, CA, USA) were spray painted flat black (2X Ultracover, Rust-Oleum Corp. Vernon Hills, IL, USA) and baked at 150 °C for 48 h. Pans containing beads with odor were used for one series of assays with the same treatment combination and then discarded.

A heated odor stimulus was not provided, because heat would change the release rates of the skin odor compounds. The odor released by a heated skin odor cue would not have been comparable to unheated odor.

### Heat Cue

A 74-mm diameter silicone heating pad (Cole-Parmer, Vernon Hills, IL, USA) was placed in a black aluminum pan and covered with glass beads to a depth of 5 mm. The surface temperature of the beads measured with a thermocouple (BAT-12, Sensorteck Inc., Clifton, NJ) was 34 °C. The power cord was run through an incision in the bottom of the aluminum pan. The heating pad was not visible from above.

### Visual Cue

To add a visual cue, we cut a white tissue (Kimwipe®) into an annulus with a 75 mm outer diameter and a 55 mm inner diameter and placed it atop the beads. The resulting 55-mm-inner black circle was similar in size to visual cues used in some mosquito traps (Bidlingmayer 1994) and wind-tunnel studies (van Breugel et al. 2015). To address concerns that the white Kimwipe® was too reflective, the light gray annuli were cut out of construction paper (Staples Pastel Gray, Staples Inc. Framingham, MA, USA) that was baked for 4 days at 200 °C. To create a facsimile of the visual cue used by van Breugel et al. (2015) an IR-filter (Wratten 2 No. 87, Eastman Kodak Company, Rochester, NY, USA) circle was placed atop a rectangle of gray construction paper that covered half of the bead-filled pan. The inner circles of both annular visual cues were 55 mm in diameter. This matched the diameter of our skin odor cue.

### Visual Cue Appearance to the Mosquitoes

*Aedes aegypti* eyes have a minimum resolvable angle of 12.3 °, allowing them to discern an object approximately the size of human-height from ~ 7 m away (Muir et al. 1992). Depending on the angle of approach, the black inner circle of our white annulus should be discernible up to 26 cm away. However, because the inner and outer edges of the annulus present ellipses that are narrower from certain angles of approach, the maximum discernible distance varies based on angle of approach. The annulus may be detectable to the mosquito beyond these ranges but would be visualized as shades of gray on several ommatidia. Despite the presumed detectability of the visual cue, we observed few landings on unheated visual cues.

The visual cues obscured mosquitoes passing over them, which prevented the use of computer vision. Additionally, when a mosquito flew over the annulus and was not visible on the inner beads nor the other side, it was assumed to have landed on the paper and was scored.

### Assay-Pairing Strategy

We conducted two sets of assays daily. The first was a two-choice assay in which skin odor was presented alongside another cue (i.e., heat, visual, or heated-visual cue). The second assay, a single-choice assay, presented the same non-odor cue without a competing odor stimulus. This latter assay served as a reference for determining the extent to which mosquitoes are attracted to visually indistinct heat, visual, and heated visual cues. The order of the assays was alternated daily. Mosquitoes used on a given day were from the same emergence cohort. Each treatment combination was tested across a set of several days.

### Cue Presentation

Treatments were presented on a 5-mm layer of black glass beads in a 32.5 by 26.5 cm black aluminum pan. The visually homogeneous layer of beads allowed heat or skin odor to be presented independent of a co-located visual cue (Fig. 3D).

In all trials, a 100 ml/min plume of 4% CO_2_ and 96% tank air was introduced at the same height as the mosquito release cage to simulate the presence of an upwind vertebrate host. This plume was separated from all cues presented on the tunnel floor (Figs. 1B and 2).

### Bioassay Procedure

Five female *Ae. aegypti* were transferred to release cages 3 h before assays. The mosquitoes were allowed one minute to acclimate to the wind tunnel. The release cage was opened at the upwind end and the mosquitoes were allowed to fly freely under video observation for 6 min.

### Data Collection

Landings were scored when a mosquito stopped movement on one of the 55-mm-diameter cues. Individual mosquitoes are visually indistinguishable and potentially could land multiple times on one or both treatments. All landings on the cues during the 6-minute observation period were scored manually with BORIS v.5.1.0 (Friard and Gamba 2016). All data manipulation and statistical tests were conducted using R v.3.5.0 (R Core Team 2013; RStudio Team 2015). The times of the following events were recorded: opening of the release cage, and latencies of takeoff, landing upon, and departure from either of the two cue presentation areas. This allowed for the calculation of the duration of each landing. Flying mosquitoes were indistinguishable on an individual basis. Therefore, “take off” considers only the first mosquito to take off.

### Statistical Analysis

The counts on each treatment type were summed by trial and entered into a Wilcoxon signed-rank test. This test treated each trial as a block. Because only one odor source volunteer was used each day, this was *de facto* blocking by odor source. This statistical blocking also accounts for daily variations in volunteer odor.

The Wilcoxon signed-rank test has an assumption that mosquito choices are independent events. This means that an animal choosing one option cannot subsequently choose another. Our free-flight wind tunnel allows a mosquito to land on one cue presentation area, take off, and land again on the other cue presentation area. The few observed occurrences of landing on both cues were noted and not used in our analyses.

As we cannot keep track of individual mosquitoes and the camera field of view does not fully cover the assay chamber, a few mosquitoes may have taken off from one cue presentation area, flown out of field of view or into glare, and then landed on the other area. Because only a handful of mosquitoes landed on both cue areas within the camera field of view, we believe that only a small percentage of mosquitoes left the field of view and returned to a different cue presentation area. Therefore, to meet the independence assumptions of the Wilcoxon signed-rank test, 5% of landings were trimmed from trials in which both cue presentation areas elicited at least one mosquito landing. A 5% trim was chosen because we believe the percentage of mosquitoes that completed two landings on different cues without being observed and discounted was less than 5%. This trimming was conducted out of an abundance of caution and the trim level was selected *a priori*. To be invalid, more than one in twenty landings must have been by a mosquito that conducted an improbable maneuver. It is analogous to the “trimmed mean” method described by Tukey and McLaughlin (1963) which is still in common usage (Zhou et al. 2014). Conducting assays of single mosquitoes would have eliminated this problem but would have quintupled the number of assays required from 520 to 2600. We have included Wilcoxon signed-rank test outputs generated without the use of this trimming. None of the probability values change across the 0.05 threshold of significance (Table S1).

## Results

We show that a small source of human skin odor in a larger homogeneous visual background elicited far more mosquito landings than a similarly sized heat (p < 0.001), visual (p < 0.001), or visually distinct heat cue (p < 0.001). The CO_2_ plume was vertically separated from the visual, heat and skin-odor cues, so that at the moment of landing, the choice of stimulus was independent of concurrent sensing of fluctuations in CO_2_ concentration.

Our study shows that *Ae. aegypti*, following an encounter with an above-ambient concentration of CO_2_ as occurs upon approach to a human, uses skin odor to pinpoint a landing site suitable for a blood meal. We found that this diurnal mosquito chooses skin odor over heat, and heat over visual cues.

The y-axes of the plots are adjusted by the number of mosquitoes flown. This makes the bar graphs visually comparable within lettered sections. This concession for accuracy does make it harder to view the low numbers of landings on some treatments. A table of the exact data is available (https://github.com/bendemasisumner/Primacy-of-Human-Odors-Aedes-aegypti).

**Fig. 4:**
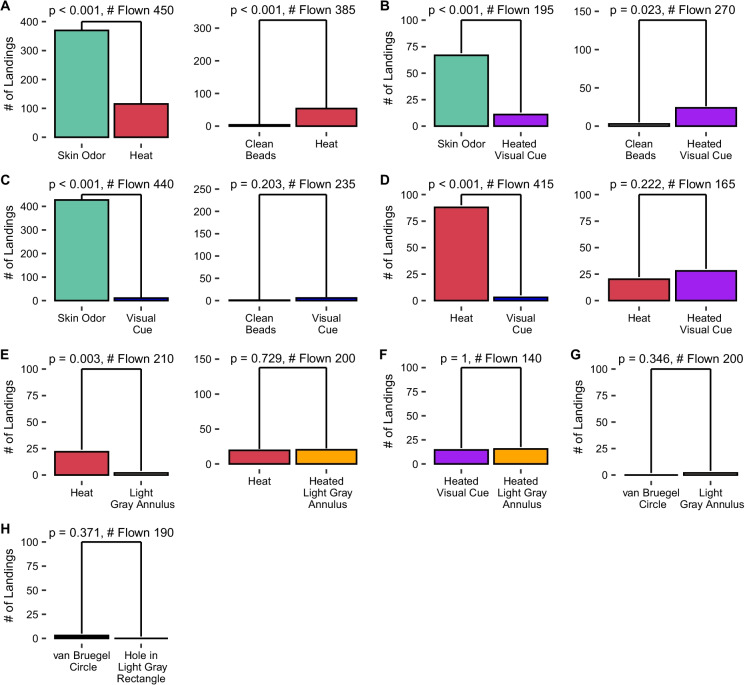
Landing counts within a lettered box were by mosquitoes from the same rearing cohorts and were flown on the same set of days. Y-axes are adjusted, within each set of days, in proportion to number assayed. This means the bar graphs are comparable within each lettered section. The calculated probabilities (p-values) above the bars show differences within trial type, not between types of trials (Wilcoxon signed-rank test)

Mosquitoes landed on skin odor far more frequently than any other stimulus. The heated visual cue and visually indistinct heat cue elicited intermediate numbers of landings. Although the landings are not statistically comparable across groups, the unheated visual cue elicited the fewest landings (Fig. 4C).

Of 450 mosquitoes given a choice of skin odor or a heat cue, there were 369 landings on skin odor and 115 landings on the heat cue (p < 0.001). On the same days, 385 mosquitoes were assayed with unheated clean beads versus a heat cue; there were 4 landings on the unheated clean beads and 54 landings on the heated beads (p < 0.001) (Fig. 4A).

Of 195 mosquitoes assayed with skin odor versus a heated visual cue, there were 66 landings on skin odor and 10 landings on the heated visual cue (p < 0.001). On the same days, 270 were assayed with unheated clean beads and a heated visual cue. There were only 3 landings on the unheated clean beads while there were 24 landings on the heated visual cue (p = 0.023) (Fig. 4B).

We assayed 440 mosquitoes with skin odor versus a visual cue. The skin odor elicited 427 landings while there were 10 landings on the visual cue (p < 0.001). On those same days, 235 mosquitoes were assayed with unheated clean beads and a visual cue. There was only one landing on the unheated clean beads and 6 landings on the visual cue [not significantly different (p = 0.203) (Fig. 4C)].

We assayed 415 individuals with a heat cue and a visual cue. The heat cue elicited 88 landings while the visual cue elicited only 3 landings (p < 0.001). On those same days, 165 individuals were presented with a heat cue which elicited 20 landings and a heated visual cue which elicited 28 landings [not significantly different (p = 0.222) (Fig. 4D)].

A light gray annulus alone elicited fewer landings than a visually indistinct heat cue (p = 0.003), and a heated light gray annulus did not elicit any more landings than a heated visually indistinct heat cue (p = 0.729) (Fig. 4E).

To verify that our visual cue was not “repellent,” we compared a heated white annulus to a heated light gray annulus. There was no significant difference in landing (Fig. 4F). We found no difference in the number of landings elicited by our light gray annulus against our copy of the IR-filter used by by van Bruegel et al. (2015) (Fig. 4G). We tested our black glass bead visual cue against an IR-filter as used by van Bruegel et al. (2015). Again, there was no difference in landing (Fig. 4H). In this case we provided a neutral gray background that covered one side of the pan. None of unheated visual cues elicited significantly more landings than clean beads.

**Fig. 5:**
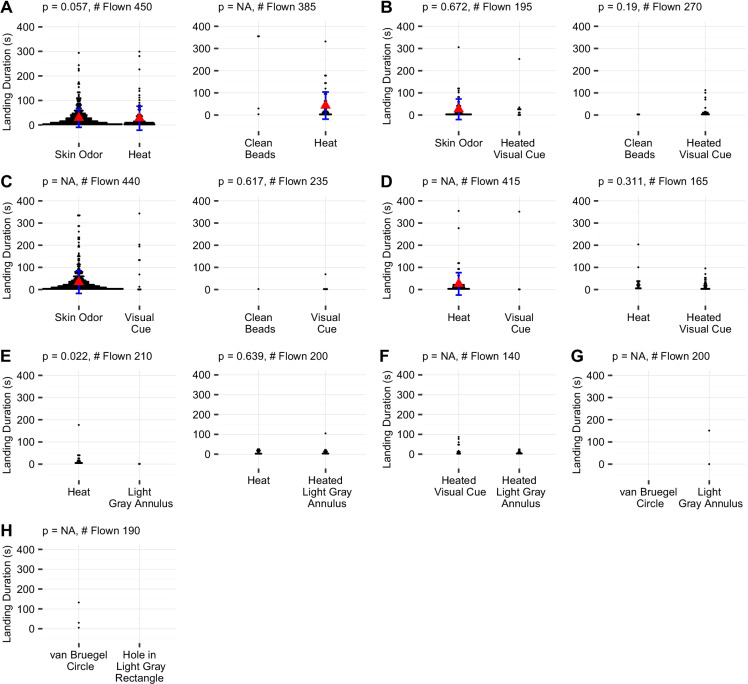
Landing durations within a letter were by mosquitoes flown on the same days. The p-values in the dot plots show differences within trial type, not between the two types of trials in each lettered section (Kruskal-Wallis test, no correction). The x-axis in each graph is the treatment presented. The lateral displacement of the dots is to ensure that the dot does not overlap with its neighbors. The more landings had a given duration, the more lateral displacement was graphically required. The red triangles indicate the median. A p-value of “NA” indicates an insufficient sample size

To address the possibility that differences between our visual cue and those those used by other researchers were leading to a false negative, we replicated the black disk used by van Bruegel et al. (2015), and presented it, 5 mosquitoes at a time, to 390 mosquitoes. Only two landed on it, although many flew near the disk. We also tested a black circle of beads exposed through a hole cut into gray paper. This provided a small black cue in a larger area of neutral color yet still did not elicit landings (Figs. 4H and 5H). This means that all of or visual cues provided on the floor, which were approximately similar to those tested in the literature, failed to elicit substantial mosquito landing.

### Landing Durations

Most pairs of cues, even those in which one elicited vastly more landings than the other, did not have significantly different landing durations (Fig. 5A, B, and C). Some of these were not statistically comparable due to insufficient sample size for at least one cue (Fig. 5C, G, and H). There were no statistical differences between heat versus heated visual cues (Fig. 5D and G). This was mirrored by the lack of significant difference between durations of landings on heat versus light gray annulus (Fig. 5E).

## Discussion

Host-seeking in mosquitoes is traditionally understood to be a series of sequential orientation maneuvers. As a mosquito approaches a host, it encounters cues produced by the host, that release a series of discrete maneuvers (Cardé and Willis 2008; Cardé and Gibson 2010; Lacey and Cardé 2011; van Breugel et al. 2015; Cardé 2015). The maneuvers include flight upwind along plumes laden with host cues and orientation toward visual cues. In this sequential-distance model, the mosquito is presumed to encounter progressively more host-specific cues, on the assumption that additional cues that are host-specific become detectable as the mosquito closes the distance to the host. The sequential-distance model does not explain choice when more than one cue is available. *Aedes aegypti* land on a heat source when CO_2_ is elevated above background, but rarely on the CO_2_ source itself (Lacey et al. 2014; McMenamin et al. 2014). The malaria mosquito *Anopheles coluzzii* landed on a nylon mesh patch imbued with skin odor rather than a CO_2_ source provided several centimeters away. However, the mosquitoes landed on the mesh only when a plume of elevated CO_2_ concentration was present (Webster et al. 2015). In both species (Lacey et al. 2014; McMenamin et al. 2014; Webster et al. 2015), the mosquitoes choose to land on the skin odor or heat over the CO_2_ source.

Other versions of the sequential-distance model suggest that the pairing of cues in addition to the distance between a mosquito and its host also plays a part in determining the mosquito’s response. For instance, one cue may lower the response threshold for another such as skin odor or visual cues (Dekker et al. 2005; van Breugel et al. 2015; Cardé 2015). We propose that in addition to host seeking based on pairs of cues that are detectable a given point, *Ae. aegypti* has an innate hierarchy of cue preference.

Mosquitoes track odor plumes upwind toward their source by optomotor aenemotaxis (Kennedy 1940; Dekker and Cardé 2011). How far mosquitoes are able to track odor plumes is not precisely known. Using rings of traps around a source of ~ 1 L/min CO_2_, which is equivalent to ~ 4 sedentary adult humans (Snow 1970; Schreck et al. 1972) captured mosquitoes from many genera, including *Aedes.* Of these, 92% were captured within 18 m of the CO_2_ source, suggesting that a human CO_2_ plume would be attractive to mosquitoes within less than ~ 18 m. This finding has a source of bias, as distant traps cover a smaller angle from the CO_2_ source. This may have provided an underestimate of the distance of attraction.

After following a plume of CO_2_, the sequential-distance model assumes that host-seeking *Aedes* mosquitoes orient toward visual cues (Cardé and Gibson 2010; van Breugel et al. 2015). The eyes of *Ae. aegypti* have a minimum resolvable angle of 12.3 °, allowing them to discern a human-sized object from ~ 7 m away (Muir et al. 1992). The black inner circle of our white annulus would be discernible up to 26 cm away, depending on the angle of approach. However, we observed very few landings on unheated visual cues (Fig. 4C, D, E, G, and H). Although the visual cue provided was obviously different from that provided by a human, it was similar to those presented by other researchers (van Breugel et al. 2015).

The scarcity of landings on our unheated visual cues contrasts with the finding of van Bruegel et al. (2015) that *Ae. aegypti* spent more time flying near a visual than a heat cue. However, they did not report whether the mosquitoes that flew within “an 8 × 8 × 4 cm volume above and downwind” of the black disk, made out of near-infrared transparent plastic, actually landed on it. Others have observed that *An. coluzzii* often fly near visual cues without landing on them (Hawkes and Gibson 2016).

Liu and Vosshall (2019) found that magnetically tethered *Ae. aegypti* orient toward black vertical stripes, which would support that visual cues are important prior to landing. They also quantified mosquito occupancy, i.e., landings and remaining landed, after landing on an unheated visual cue. Although a vertically oriented black dot elicited more mosquito occupancy than the surrounding white paper, it was less than half of mosquito occupancy of heat without a visual cue.

Our results affirm that even in the presence of elevated CO_2_, visual cues elicit few landings. Few mosquitoes landed on the small, clean, unheated visual cues. A close mimic of human visual cues would have been preferred but would have interfered with the presentation of other cues. The minimal response to the alternative visual cues is shown in Fig. 4H and 5H. Because landing is a prerequisite for blood feeding and therefore pathogen transmission, it is an important diagnostic measure of mosquito host-seeking behavior.

In studies of insect landing in which stereotyped maneuvers precede coming into contact with the substrate, insects were provided with a distinct visual cue. Leg extension, body turns (saccades) and pitch change (van Breugel and Dickinson 2012) are triggered by objects covering an expanding portion of the insect visual field. Honey bees can maintain a fixed angle relative to a visual cue in order to execute a smooth descent path toward and landing upon the cue (Srinivasan and Zhang 1997; Srinivasan et al. 2000). In contrast to those studies with prominent visual cues, we presented heat and skin odor without co-located visual cues. The visual cues available to the mosquito for optomotor feedback were lateral and above the mosquito (Fig. 2A). Our mosquitoes still executed landings, using a mechanism different from those previously studied.

In addition to following odor plumes, *Ae. aegypti* at close range can follow convective heat plumes. *Aedes aegypti* landed frequently on a nylon mesh cone placed 5 cm above a 43.3 °C black billiard ball but failed to land when the convection was interrupted by a long-wave, infrared-transparent KRS-5 filter (Peterson and Brown 1951). The tips of the antennae of *Ae. aegypti* are equipped with neurons housed in coeloconic sensilla that exhibit phasic shifts in response to air temperature changes as small as 0.05 °C (Davis and Sokolove 1975). The human-temperature heating pad in our assay produced a plume 0.2 °C above ambient ~ 30 cm downwind. Therefore, the heat plume could have been detectable to the mosquitoes throughout a large swath of the wind tunnel and yet they still preferred to land on a source of skin odor.

The distance at which skin odor plumes are detectable to a mosquito in the field is unclear. Dekker and Cardé (2011) found that skin odor, supplied by an odor stream from an enclosed arm ~ 1 m upwind, readily elicited upwind flight of *Ae. aegypti*. However, when the skin odor plume was diluted five-fold, it elicited proportionally fewer flights. When a plume of CO_2_ was added, the mosquitoes were “instantly sensitized” to the diluted skin odor and surged upwind (Dekker and Cardé 2011). Such sensitization clearly could have occurred in our trials, and also would have contributed to landing on a skin odor patch in *An. coluzzii* (Webster et al. 2015).

Skin odor is thought to be the cue that anthropophilic mosquitoes use to tell humans apart from other endothermic vertebrate hosts (Gouck 1972; Takken et al. 1997; Pates et al. 2001; Dekker et al. 2001, 2002; Besansky et al. 2004; McBride 2016; DeGennaro et al. 2013) found that *orco* mutant *Ae. aegypti* lose their strong preference for human odor over that of guinea pig in a cage assay. By lacking *orco*, the olfactory coreceptor, these mosquitoes lost the function of all of their olfactory receptors. Anopheline mosquitoes (Ribbands 1946) and *Ae. aegypti* (Trpis and Hausermann 1978) also appear to use human scent for house-entering, which can occur well before biting. Once inside a human dwelling, host seeking could be triggered by a fluctuating concentration of CO_2_ (Dekker and Cardé 2011). Our findings show that human skin odor not only provides a human-specific cue, but also provides a direct host-seeking and landing cue.

Landing durations were contrasted with a Kruskal-Wallis test. Because the number of landings on a cue is, by definition, the sample size of landing duration, the differences in landing counts on different cues may have limited the statistical power of our test and may explain some of the lack of significant differences between landing durations. This is not unexpected, because the experiment was designed to show a difference in the number of landings on different cues. Landing and remaining landed are separate behavioral categories. Landing duration is a potential measure of cue salience. Mosquitoes have chemoreceptors on their tarsi (Sparks et al. 2013) and labella (Saveer et al. 2018). Once a mosquito’s tarsi or labella contact beads treated with skin odor, the mosquito might detect non-volatile chemicals such as amino acids.

## Conclusions

Here we establish that following exposure to a plume of CO_2_, heat stimulus, and a heated visual cue, all evoke at least some orientation and landing. However, the use of a choice bioassay allowed these cues to be ranked in a valance hierarchy. Naïve *Ae. aegypti* had a high preference for landing on a skin odor source; heated cues were the second most effective cue for eliciting landing, whereas the unheated visual cues elicited virtually no landings. This demonstrates that *Ae. aegypti*, a day-biting mosquito, are able to locate and land on skin odor and heat without a co-located visual cue. The order, primacy, and interaction of cues used by *Ae. aegypti* and other mosquitoes during host finding in the field remain to be firmly established. However, our findings suggest that *Ae. aegypti* track and land on cues based on an innate hierarchy. This hierarchy appears to rank cue types in descending order of human specificity. The primacy of skin odor contrasts with the view that this diurnal mosquito relies primarily on vision to find and potentially land on a host following detection of a fluctuating concentration of CO_2_.

## Supplementary Information

Below is the link to the electronic supplementary material.

ESM 1 (DOCX 25.6 KB)

## Data Availability

GitHub link to follow.

## Code Availability

GitHub link to follow.
