# Supplementary material for: Primacy of Human Odors Over Visual and Heat Cues in Inducing Landing in Female Aedes aegypti Mosquitoes
Source: J Insect Behav. 2022 May 23;35(1-3):31–43. doi: 10.1007/s10905-022-09796-2 (PMC9276619; doi:10.1007/s10905-022-09796-2)
Supplement: Supplementary file 1 — (DOCX 25.6 KB) [file 10905_2022_9796_MOESM1_ESM.docx]

Primacy of Human-Odors over Visual and Heat Cues in Inducing Landing in Female *Aedes aegypti* Mosquitoes

| **Treatment** | **Control** | **Treatment landings after trim** | **Control landings after trim** | **p-value after trim** | **Treatment landings no trim** | **Control landings no trim** | **p-value without trim** |
| --- | --- | --- | --- | --- | --- | --- | --- |
| Skin Odor | Heat | 369.6 | 115.2 | 0.00000176 | 376 | 118 | 0.00000156 |
| Clean Beads | Heat | 4 | 54 | 0.00012234 | 4 | 54 | 0.00012234 |
| Skin Odor | Heated Visual Cue | 66.9 | 10.95 | 0.00030519 | 67 | 11 | 0.00029541 |
| Clean Beads | Heated Visual Cue | 3 | 24 | 0.02320554 | 3 | 24 | 0.02320554 |
| Skin Odor | Visual Cue | 427.25 | 10.55 | 0 | 430 | 11 | 0 |
| Clean Beads | Visual Cue | 1 | 6 | 0.20309179 | 1 | 6 | 0.20309179 |
| Heat | Visual Cue | 88 | 3 | 0.00000054 | 88 | 3 | 0.00000054 |
| Heat | Heated Visual Cue | 20.25 | 28.05 | 0.22150949 | 21 | 29 | 0.25334915 |
| Heat | Light Gray Annulus | 22 | 2 | 0.00284787 | 22 | 2 | 0.00284787 |
| Heat | Heated Light Gray Annulus | 19.45 | 20.4 | 0.72859961 | 20 | 21 | 0.88378219 |
| Heated Visual Cue | Heated Light Gray Annulus | 14.55 | 15.6 | 1 | 15 | 16 | 0.96344182 |
| van Bruegel Circle | Light Gray Annulus | 0 | 2 | 0.34577859 | 0 | 2 | 0.34577859 |
| van Bruegel Circle | Hole in Light Gray Rectangle | 3 | 0 | 0.37109337 | 3 | 0 | 0.37109337 |

Table S1 Landing counts and probability values on the left side are the same as those shown in Fig. 4 although they are not abbreviated here. The landing counts and probability values on the right side were produced with the same Wilcoxon Rank Sum Test, but without the removal of 5% of the landings from trials with landing on both cues. The trimming procedure did not change the significance (p **<** 0.05) of any assay result.
